# Mesenchymal stromal cells as vehicles of tetravalent bispecific Tandab (CD3/CD19) for the treatment of B cell lymphoma combined with IDO pathway inhibitor d-1-methyl-tryptophan

**DOI:** 10.1186/s13045-017-0397-z

**Published:** 2017-02-23

**Authors:** Xiaolong Zhang, Yuanyuan Yang, Leisheng Zhang, Yang Lu, Qing Zhang, Dongmei Fan, Yizhi Zhang, Yanjun Zhang, Zhou Ye, Dongsheng Xiong

**Affiliations:** 1 State Key Laboratory of Experimental Hematology, Institute of Hematology and Hospital of Blood Diseases, Chinese Academy of Medical Science and Peking Union Medical College, Tianjin, 300020 People’s Republic of China; 2 Central Hospital of Karamay, Karamay, Xinjiang 834000 People’s Republic of China

**Keywords:** MSCs, Bispecific antibodies, CD3, CD19, d-1-methyl-tryptophan, B cell lymphoma

## Abstract

**Background:**

Although blinatumomab, a bispecific T cell engaging antibody, exhibits high clinical response rates in patients with relapsed or refractory B-precursor acute lymphoblastic leukemia (B-ALL) and B cell non-Hodgkin’s lymphoma (B-NHL), it still has some limitations because of its short half-life. Mesenchymal stromal cells (MSCs) represent an attractive approach for delivery of therapeutic agents to cancer sites owing to their tropism towards tumors, but their immunosuppression capabilities, especially induced by indoleamine 2,3-dioxygenase (IDO), should also be taken into consideration.

**Methods:**

Human umbilical cord-derived MSCs (UC-MSCs) were genetically modified to secrete Tandab (CD3/CD19), a tetravalent bispecific tandem diabody with two binding sites for CD3 and two for CD19. The tropism of MSCs towards Raji cells in vitro was determined by migration assays, and the homing property of MSCs in vivo was analyzed with firefly luciferase-labeled MSCs (MSC-Luc) by bioluminescent imaging (BLI). The cytotoxicity of T cells induced by MSC-secreting Tandab (CD3/CD19) was detected in vitro and in vivo in combination with d-1-methyl-tryptophan (D-1MT), an IDO pathway inhibitor.

**Results:**

The purified Tandab (CD3/CD19) was functional with high-binding capability both for CD3-positive cells and CD19-positive cells and was able to induce specific lysis of CD19-positive cell lines (Raji, Daudi, and BJAB) in the presence of T cells. Additionally, results from co-culture killing experiments demonstrated that Tandab (CD3/CD19) secreted from MSCs was also effective. Then, we confirmed that D-1MT could enhance the cytotoxicity of T cells triggered by MSC-Tandab through reversing T cell anergy with down-regulation of CD98 and Jumonji and restoring the proliferation capacity of T cells. Furthermore, MSC-Luc could selectively migrate to tumor site in a BALB/c nude mouse model with Raji cells. And mice injected with MSC-Tandab in combination with D-1MT significantly inhibited the tumor growth.

**Conclusions:**

These results suggest that UC-MSCs releasing Tandab (CD3/CD19) is an efficient therapeutic tool for the treatment of B cell lymphoma when combined with D-1MT.

**Electronic supplementary material:**

The online version of this article (doi:10.1186/s13045-017-0397-z) contains supplementary material, which is available to authorized users.

## Background

During the past two decades, therapeutic antibodies have started to make major contributions to the treatment of B cell malignancies. In 1997, rituximab (a genetically engineered monoclonal chimeric antibody directed against the CD20 B cell antigen) was approved by the US Food and Drug Administration (FDA) for the indication of follicular lymphoma and low-grade B cell non-Hodgkin’s lymphoma (B-NHL) [1]. However, cancer relapse and metastasis often occurred after medical intervention with CD20-based therapy in patients with follicular lymphoma and acute lymphocytic leukemia [2]. Thus, it is absolutely critical to develop new therapeutic regimens to overcome these challenges.

T cells are the most potent tumor-killing effector cells, but they cannot be recruited by conventional antibodies. However, several bispecific antibodies (bsAbs) that recruit T cells have been developed [3], which may have the potential to circumvent this problem. To date, the most promising for the therapeutic application of this approach is blinatumomab, a tandem single-chain variable fragment (scFv) bsAb in a bispecific T cell engager (BiTE) format targeting CD19/CD3, which was approved by the FDA for the treatment of B-precursor acute lymphoblastic leukemia (B-ALL) [4]. Although blinatumomab has impressive efficacy in the clinic and exhibits high clinical response rates in patients with relapsed or refractory B-ALL and B-NHL [5, 6], it still has some limitations due to its low molecular weight (~55 kDa), which is below the glomerular filtration threshold [6]. Therefore, blinatumomab is administered over a 28-day continuous infusion using a mini-pump in order to maintain steady drug concentration [6], which results in inconvenience for patients and an increased possibility of treatment-related adverse event. To overcome the drawback of short half-life, Kipriyanov and his colleagues [7] firstly designed a tetravalent bispecific tandem diabody (TandAb) with two binding sites for CD3 and two for CD19. Due to the TandAb’s large molecular weight (~105 kDa), it is not subject to glomerular filtration. In addition, the molecule exhibits stability properties with a half-life ranging from 18.4 to 22.9 h after intravenous administration in mice [8].

Most of therapeutic antibodies are administrated by intravenous infusion, which results in a handful of antibodies that can reach the tumor sites. Thus, introducing an efficient and targeted delivery system for these therapeutic antibodies may enhance the efficacy of treatment for tumors, especially for minimal residual diseases (MRD) [9]. Mesenchymal stromal cells (MSCs) are attractive cellular vehicles for the therapy of malignant diseases as they have the ability to migrate into tumors and track microscopic metastasis [10, 11]. We have previously employed human umbilical cord-derived MSCs (UC-MSCs) as carriers for gene therapy [12, 13] because UC-MSCs are easier to isolate and expand, and the harvesting procedure is more consistent and yields a greater number of relevant cells than other adult and feta tissues [14]. These characteristics indicate that UC-MSC is a promising targeted delivery system with anticancer agents for a variety of cancers.

However, a study from Ribeiro and his colleagues shows that MSCs derived from different tissues possess different immunosuppression capabilities and their action varies with the immune cell type [15]. Thus, MSCs are actually a double-edged sword when employed as carriers with agents triggering cytotoxicity of T cells for tumors. Although the exact mechanisms of MSC-mediated immunosuppression are still debated, many different factors are believed to be involved. It has been revealed that indoleamine 2,3-dioxygenase (IDO), the first and rate-limiting enzyme in the degradation of tryptophan [16], plays a role in human MSCs to regulate immunity in tumor microenvironment [17]. By depleting tryptophan locally and accumulation of its metabolites such as kynurenine and quinolinic acid, which could interact the aryl hydrocarbon receptor (AHR) on T cells, IDO seems to block the proliferation of T cells and inhibit T cell activity [18, 19]. Because IDO is induced by inflammatory cytokines such as interferon-γ (IFN-γ) which act through JAK-STAT signaling pathways on interferon stimulatory response elements (ISRE) and γ-activating sequences (GAS) in the IDO promoter [20], its expression is thought to be an endogenous feedback mechanism controlling excessive immune response [21].

In this study, we employed the TandAb platform to construct a new tandem tetravalent antibody targeting both CD3 and CD19 (Tandab (CD3/CD19)) using our own DNA sequences of scFvs. Then, we exploited the feasibility and efficacy of using genetically modified UC-MSCs which constitutively secreted Tandab (CD3/CD19) (MSC-Tandab) for the treatment of human B cell lymphoma. Our results showed that MSC-Tandab could induce specific lysis of CD19-positive Raji cells in the presence of T cells in vitro and reduce xenograft tumor growth in vivo in combination with d-1-methyl-tryptophan (D-1MT), an IDO pathway inhibitor [22].

## Methods

### Cell lines and cell culture

Human embryonic kidney cell-derived 293T cells (kindly provided by Professor Cheng Tao, PUMC) were maintained in DMEM (Invitrogen, USA) supplemented with 2 mM l-glutamine, 100 U/mL penicillin (Gibco, USA), 100 μg/mL streptomycin (Gibco, USA) and 10% FBS (Gibco, USA). The human acute T cell leukemia cell line Jurkat, human chronic myelogenous leukemia cell line K562, and human B cell lymphoma cell lines Raji, Daudi, and BJAB (Institute of Hematology and Blood Diseases Hospital, Chinese Academy of Medical Science and Peking Union Medical College, Tianjin, China) were grown in RPMI-1640 medium (Invitrogen, USA) supplemented with 2 mM l-glutamine, 100 U/mL penicillin (Gibco, USA), 100 μg/mL streptomycin (Gibco, USA), and 10% FBS. The cells were incubated at 37 °C in a humidified atmosphere containing 5% CO_2_.

### PBMC isolation

With informed consent, human peripheral blood mononuclear cells (PBMCs) were isolated from healthy volunteers. Details are provided in the Additional file 1.

### Construction of lentiviral expression vectors

Plasmids pLH-T3a*-19a (containing a cysteine at position Ser-100 of V_L_3) and pLH-19a-T3a* (containing a cysteine at position Gly-44 of V_H_3) encoding for hybrid V_L_3-V_H_19 and V_L_19-V_H_3 scFvs [23], respectively, were used for assembly of Tandab (CD3/CD19) genes. See details in Additional file 1.

### Expression and purification of Tandab (CD3/CD19)

293T cells were transfected with pLentiR-Tandab (CD3/CD19) or pLentiR-EV using Lipofectamine 2000 (Invitrogen, USA) according to the manufacturer’s protocol. After 48 h of transfection, supernatants were collected by centrifugation at 500×*g* for 10 min at 4 °C to clear 293T cells. The soluble antibodies in the supernatants were purified by 6×His-tag affinity chromatography (GE Healthcare, Sweden) according to the manufacturer’s instruction. The purified preparations were quantified with His-tag ELISA detection kit (GenScript, USA) and were used for cell-binding assays and cytotoxicity assays in vitro. In addition, the unpurified or purified Tandab (CD3/CD19) were verified by Western blot analysis.

### Cell-binding assay

The CD19-positive cell lines Raji, Daudi, and BJAB and the CD3-positive cell line Jurkat were employed for analysis of binding activity of Tandab (CD3CD19) by flow cytometry (LSRII, Becton Dickinson Bioscience, San Jose, CA). The CD19- and CD3-negative K562 cells were served as negative control. See details in Additional file 1.

### Cytotoxicity assay

All cytotoxicity assays were performed with PBMC effector cells. And PBMCs were pre-activated with 50 IU/mL IL-2 for 3 days before cytotoxicity assays. CD19^+^ cells (Raji, Daudi, and BJAB) and CD19^−^ cells (K562) were prepared as target cells. The specific lysis of target cells was detected by LDH release assay according to the manufacturer’s protocol. See details in Additional file 1.

### MSCs preparation

MSCs were isolated from human umbilical cord Wharton’s jelly (WJ) as previous described [24]. MSCs were cultured at a density of 8 × 10^3^ cell/cm^2^ in DF-12 medium (Invitrogen, USA) supplemented with 2 mM l-glutamine and 10% FBS (Gibco, USA). When cells reached 80~90% confluence, they were detached using a 0.125% trypsin/1 mM EDTA solution and re-seeded using the same growth medium for subsequent passages. For all experiments, early passages MSCs (3P to 5P) were used.

### Production of lentivirus

The lentiviral particles carrying Tandab (CD3/CD19) gene were packaged according to the SBI’s protocol. See details in Additional file 1.

### Transduction of MSCs and viability of transduced MSCs

The transduction of MSCs was performed as previously reported [12]. And viability of transduced MSCs was detected by MTT assays. See details in Additional file 1.

### Immunophenotype profile and tri-lineage differentiation of MSCs

MSCs and transduced MSCs (including MSC-EV and MSC-Tandab) were trypsinized (0.125% trypsin-EDTA) and washed twice with PBS, then incubated with APC-labeled anti-human CD73, CD90, CD105, CD14, CD19, CD34, CD45, and HLA-DR (all from BD Biosciences) for 30 min. After washing with PBS, the expression level of these molecules was determined by flow cytometry.

To test the in vitro differentiation ability, MSCs or transduced MSCs were cultured in adipogenic, osteogenic, and chondrogenic differentiation medium, respectively. For adipogenic differentiation, the MSCs were maintained in medium containing 1 mM dexamethasone, 500 μM IBMX, 10 μg/mL insulin, and 60 μM indomethacin (all from Sigma). Three weeks later, the cells were fixed and stained with Oil Red O (Sigma). For osteogenic differentiation, cells were cultured in IMDM (Gibco) supplemented with 10% FBS, 100 nM dexamethasone, 50 μg/mL ascorbic acid, and 10 μM β-glycerophosphate (all from Sigma) for about 3 weeks. At the end of incubation, the cells were assayed by Alizarin Red S (Sigma) staining for calcium deposition. To induce chondrogenic differentiation, MSCs were maintained in medium with 100 nM dexamethasone, 50 μg/mL ascorbic acid, 40 μg/mL proline, 10 ng/mL TGF-β_3_, 2 mM ITS, 53.5 μg/mL lindeic acid, and 12.5 μg/mL BSA (all from Sigma). Three weeks later, the cultured cells were stained with Alcian Blue (Sigma). In addition, RNA was isolated from cells after the induction, and the expression level of differentiation-related gene was determined by real-time PCR on an ABI Prism 7500 detection system (Applied Biosystems, USA). And primers used for real-time PCR were summarized in Table 1.

**Table 1:** Primer sequences for genes in real-time PCR

Gene	Primer sequence (from 5′ to 3′)
PPAR-r	Forward: GCTGGCCTCCTTGATGAATA Reverse: TGTCTTCAATGGGCTTCACA
ADIPOQ	Forward: TGGTCCTAAGGGAGACATCG
	Reverse: TGGAATTTACCAGTGGAGCC
RUNX2	Forward: CTCACTACCACACCTACCTG
	Reverse: TCAATATGGTCGCCAAACAGATTC
BGLAP	Forward: GGCGCTACCTGTATCAATGG
	Reverse: TCAGCCAACTCGTCACAGTC
SOX9	Forward: AATGGAGCAGCGAAATCAAC
	Reverse: CAGAGAGATTTAGCACACTGATC
COL2A1	Forward: GGCAATAGCAGGTTCACGTACA
	Reverse: CGATAACAGTCTTGCCCCACTT
IDO	Forward: GCCCTTCAAGTGTTTCACCAA
	Reverse: CCAGCCAGACAAATATATGCGA
CD98	Forward: GCTGCTGCTCTTCTGGCTC
	Reverse: GCCAGTGGCATTCAAATAC
Jumonji	Forward: GCTCAGGACTTACGGAAACA
	Reverse: TGTGGTTGACAGCGGAACTG
GAPDH	Forward: GGTCTTACTCCTTGGAGGCCATGT
	Reverse: ACCTAACTACATGGTTTACATGTT

### In vitro and in vivo MSC migration assay

In vitro and in vivo MSCs migration assays were performed as previously reported [12, 25]. Details are provided in Additional file 1.

### In vitro co-culture killing experiments

To assess the bioactivity of Tandab (CD3/CD19) secreted by MSCs, a co-culture system using transwell plates with 0.4-μm-pore membrane was established. The specific lysis of target cells was determined by FACS analysis according to the “calcein-loss” method [26]. Briefly, MSC-Tandab, MSC-EV, or MSCs were seeded into 6-well culture plates at a density of 1 × 10^5^ cells per well and incubated for 72 h. Then, Raji cells labeled with calcein-AM (Sigma, USA) and pre-activated PBMCs were added to the equilibrated inserts at an E:T ratio of 10:1. After co-cultured for 24 h, the cells in the inserts were harvested to be detected by flow cytometry. The expression of activation surface markers CD69 and CD25 of T cells was detected by flow cytometry in the same conditions of co-culture system with unlabeled Raji cells. And the supernatants in the inserts were collected for the assay of cytokines produced in the co-culture system, including IL-2, IFN-γ, and TNF-α using ELISA kits (R&D system, USA).

Because of expression of IDO in MSCs induced by IFN-γ in the co-culture system, we performed a co-culture experiment for a longer time in absence or presence of D-1MT (Sigma-Aldrich, USA), which is an IDO pathway inhibitor. As mentioned above, MSC-Tandab, pre-activated PBMCs, and Raji cells were added respectively into the co-culture system with or without 1 mM D-1MT and co-cultured for 24, 48, and 72 h. Cells in the upper chamber were harvested at the indicated time points. And the residual Raji cells were determined by flow cytometry with FITC-conjugated anti-CD19 antibodies. The supernatants were harvested for the measure of kynurenine, a metabolite of tryptophan in the IDO pathway. Repetitive wells were set for detection of CD98 and Jumonji in the messenger RNA (mRNA) level at different time points (24, 48, and 72 h) by real-time PCR. In addition, the proliferation of T cells in the co-culture system was detected by BrdU Flow Kit (BD Bioscences). Details are provided in Additional file 1.

### Inducible expression of IDO in MSCs

MSCs were seeded into 6-well culture plates at a density of 1 × 10^5^ cells per well in the absence or presence of 20 ng/mL of recombinant human IFN-γ (R&D system, USA). After incubation for 48 h, expression of IDO in the level of mRNA or protein upon treatment with IFN-γ was verified by real-time PCR and Western blot analysis.

### Detection of kynurenine

Because IDO catalyzes the metabolism of tryptophan in the kynurenine pathway, the activity of IDO was determined by spectrophotometric assay for kynurenine [17] in supernatants from co-culture system and cultures of MSCs with or without exogenous IFN-γ stimulation. See details in Additional file 1.

### Cell viability assay

Cell viability assay was performed by MTT assay (Sigma-Aldrich, USA). See details in Additional file 1.

### Western blot analysis

The expression of specific protein was detected by Western blot analysis. See details in Additional file 1.

### Real-time PCR

Total RNA was extracted from corresponding cells using Trizol reagent (Invitrogen, USA) following the manufacturer’s protocol. The complementary DNA (cDNA) was generated using OligdT primers and M-MLV reverse transcriptase (Invitrogen, USA) with 2 μg total RNA. Real-time PCR was performed using ABI Prism 7500 real-time PCR system (Applied Biosystems, USA), in combination with SYBR Green (Takara, Dalian, China). Specific primers for each gene (Table 1) were selected using Primer Express (Applied Biosystems, USA). Relative transcript expression was normalized to that of GAPDH mRNA.

### Growth inhibition of B cell lymphoma xenografts in vivo

All animal studies were performed in accordance with the guidelines under the Animal Ethics Committee of the Institute of Hematology and Hospital of Blood Diseases, Chinese Academy of Medical Sciences and Peking Union Medical College. Raji cells (2 × 10^7^ cells per mouse) were implanted subcutaneously into the right flank of each BALB/c nude mice (female, 5–6 weeks of age; PUMC, China) 1 day after the application of total body irradiation (300 cGy). One week later when tumor size reached 100–200 mm^3^, the mice were treated intravenously with MSC-Tandab and pre-activated PBMCs and D-1MT in the drinking water. Then, the mice were sacrificed for analysis of organ damages in the indicated time. In tumor therapy experiments, the mice were randomly divided into six groups (five mice for each group) as follows: (a) MSC + PBMC; (b) MSC-EV + PBMC; (c) MSC-Tandab + PBMC; (d) MSC + PBMC + D-1MT; (e) MSC-EV + PBMC + D-1MT; (f) MSC-Tandab + PBMC + D-1MT. MSCs (1 × 10^6^ cells per mouse) were injected intravenously at day 0, followed by pre-activated PBMCs (5 × 10^6^ cells per mouse) via the vein 2 days later, every 7 days for 2 weeks. The mice were treated with or without D-1MT (2 mg/mL in drinking water) from the beginning to the end of the treatment. At day 21 after treatment started, the mice were sacrificed by cervical dislocation under anesthesia. Then, tumor tissues were harvested and weighted for treatment evaluation.

### Statistical analysis

Data are represented as mean ± SD. Statistical analysis was performed using GraphPad Prism 6 or Microsoft Excel software. Significance was assayed by an unpaired two-tailed Student *t* test or ANOVA (*P < 0.05, **P < 0.01, ***P < 0.001).

## Results

### Design and production of Tandab (CD3/CD19)

**Fig. 1:**
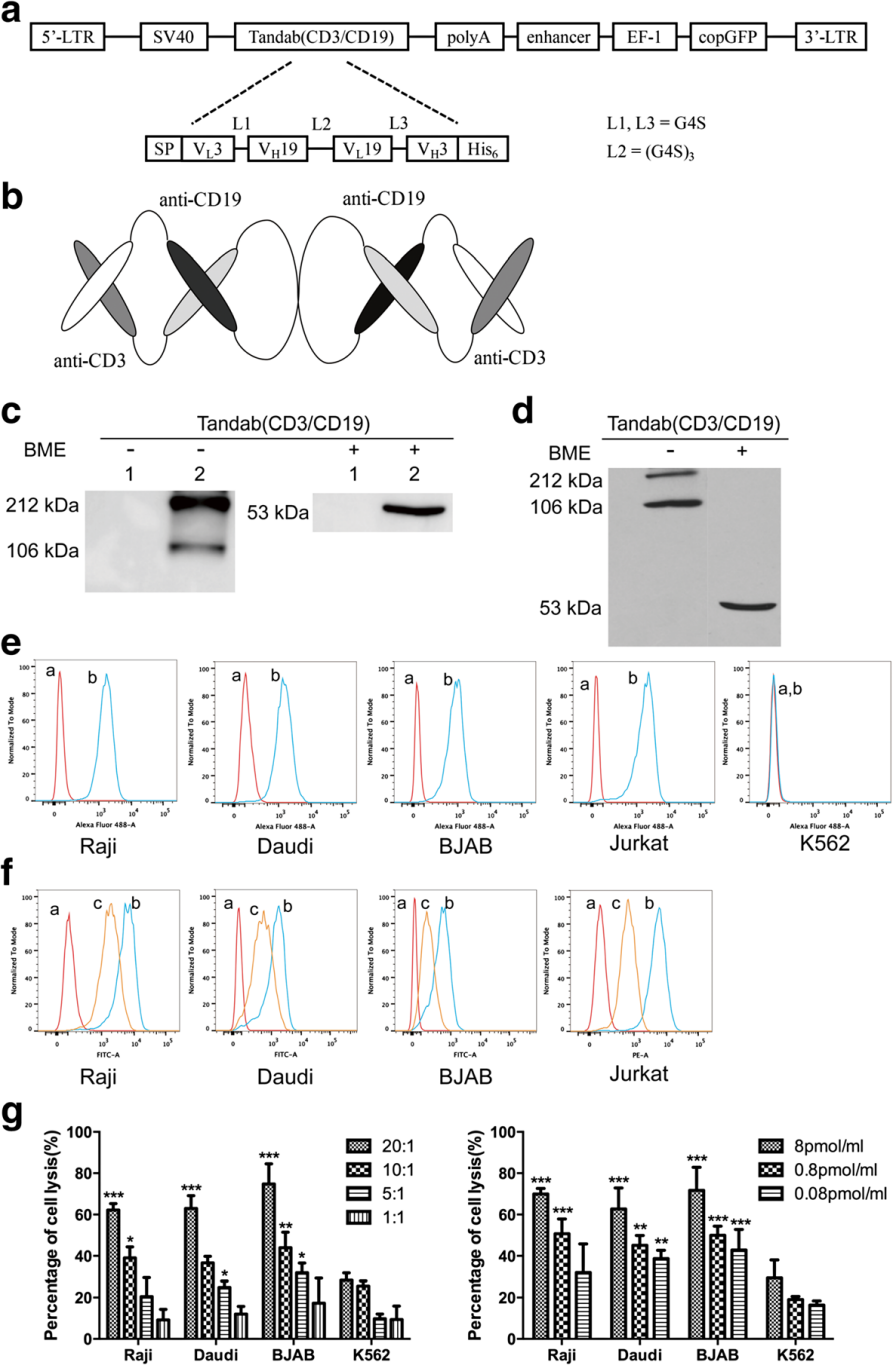
Design, production, and biological function of bispecific tetravalent tandem diabody Tandab (CD3/CD19). **a** Schematic representation of lentiviral expression vector for Tandab (CD3/CD19). LTR, long terminal repeats; SV40, SV40 promoter; SP, signal peptide, a murine kappa light-chain leader peptide; G4S, Gly-Gly-Gly-Gly-Ser residues; His_6_, hexa-histidine tag. **b** Molecular model of Tandab (CD3/CD19). Upon expression, two polypeptide gene products dimerize in a head-to-tail fashion. **c** Tandab (CD3/CD19) was determined by Western blot analysis. The supernatants from 293T cells after transfection with lentiviral expression vectors were harvested and assayed for Tandab (CD3/CD19) with or without β-mercaptoethanol (BME) using anti-His tag antibodies. Lane 1, 293T cells transfected with empty vector (negative control); lane 2, 293T cells transfected with vector expressing Tandab (CD3/CD19). **d** Western blot analysis of the purified Tandab (CD3/CD19). **e** Binding specificities of Tandab (CD3/CD19) to tumor cell lines. FACS analysis with the Tandab (CD3/CD19) on different CD19-positive B cell lines (Raji, Daudi, and BJAB), on CD3-positive Jurkat cells, and on the CD3- and CD19-negative K562 cells. *a*, negative controls with the secondary antibody anti-His-Alexa Fluor 488 alone; *b*, Tandab (CD3/CD19). **f** Competitive binding activity with FITC-conjugated HIT19a or PE-conjugated HIT3a. Cells were firstly incubated with Tandab (CD3/CD19) for 1 h at 4 °C, then incubated with FITC-conjugated HIT19a or PE-conjugated HIT3a for 30 min at 4 °C before detection. *a*, Negative controls; *b*, FITC-conjugated HIT19a or PE-conjugated HIT3a alone; and *c*, Tandab (CD3/CD19) + FITC-conjugated HIT19a or PE-conjugated HIT3a. Results are representative of three independent experiments. **g** Specific lysis of malignant target cell lines mediated by Tandab (CD3/CD19). Cytotoxicity of IL-2 pre-activated PBMCs induced by Tandab (CD3/CD19) with the same concentration (8 pmol/mL) in different effector to target (E:T) ratios ranging from 20:1 to 1:1 against CD19-positive B cell lines (Raji, Daudi, and BJAB) was detected by LDH release assay (*left panel*). Specific lysis of target cells by Tandab (CD3/CD19) with different concentrations at the same E:T ratio (20:1) was also determined (*right panel*). K562 cells were served as negative controls. *P < 0.05; **P < 0.01; ***P < 0.001 compared with the corresponding K562 group. Data shown are the mean ± SD of three independent experiments

The concept of dimerizing scFv fragments having a short peptide linker between the domains to create two antigen-binding sites pointing in opposite directions [27] was extended to single-chain molecules containing four antibody variable domains [8]. We previously constructed a CD3 × CD19 bispecific diabody comprising two hybrid scFv fragments: an anti-human CD3 V_L_ domain connected to an anti-human CD19 V_H_ domain by a short linker peptide (Gly-Gly-Gly-Gly-Ser, G4S) and anti-CD19 V_L_ connected to an anti-CD3 V_H_ by a similar linker [23]. We have now fused these hybrid svFvs into a single-chain polypeptide using a long (G4S)_3_ linker (Fig. 1a). The fusion gene fragment V_L_3-V_H_19-V_L_19-V_H_3 was inserted into a lentiviral expression vector under the control of constitutively active SV40 promoter. A hexa-histidine-tag (His_6_-tag) was added to the carboxyl terminus of the construct to aid in the detection and purification of the products. Upon expression, two polypeptide gene products dimerize in a head-to-tail fashion. These active homodimers (Tandab (CD3/CD19)) have two binding sites for CD3 and two for CD19 (Fig. 1b). To verify the formation of this gene products, Tandab (CD3/CD19) was firstly expressed in adherent 293T cells. In the culture supernatant, protein with a molecular weight of 106 kDa was detected on non-reducing status, and a 53-kDa protein was also detected under the reducing status (Fig. 1c). As shown in Fig. 1d, Tandab (CD3/CD19) maintained a dimer formation after purification in the natural state. Because of the interactions between molecules, a band at 212 kDa was exposed, which indicated that part of the products were tetramers.

### Tandab (CD3/CD19) binds specifically both to its target antigens

Binding specificities of the Tandab (CD3/CD19) were shown by flow cytometry analysis on a number of different CD19-positive B cell lymphoma cell lines, including Raji, Daudi, and BJAB, and CD3-positive Jurkat cells. No binding was detectable on chronic myelogenous leukemia cell line K562, which expresses neither CD19 nor CD3 (Fig. 1e). Furthermore, Tandab (CD3/CD19) was able to significantly prevent parental monoclonal antibodies HIT19a [28] or HIT3a [29] from binding to Raji, BJAB, Daudi, or Jurkat cells, respectively, in a competitive binding assay (Fig. 1f).

### Tandab (CD3/CD19) mediates specific target cell lysis in combination with PBMCs

To investigate the tumor lysis mediated by Tandab (CD3/CD19) in the presence of activated human T cells, a nonradioactive cytotoxicity assay was performed. For this purpose, a panel of CD19-positive cell lines of B cell lymphoma was used as targets with PBMC: target cell ratios ranging from 20:1 to 1:1. Tandab (CD3/CD19) was added at a concentration of 8 pmol/mL and after an 8-h reaction time, the target cell lysis was measured by LDH release described previously [30]. Although CD19-negative K562 cells were also lysed (less than 30% at the highest E:T ratio), which may result from aspecific effects, Tandab (CD3/CD19) produced significant specific lysis of CD19-positive Raji cells, as well as the Daudi and BJAB cells (Fig. 1g). Furthermore, target cells were also lysed efficiently at the same E:T ratio (20:1) with different concentrations of Tandab (CD3/CD19) (Fig. 1g). Lysis of target cells induced by Tandab (CD3/CD19) proceeded in a dose-dependent manner, in which increasing the ratio of E:T or the concentration of Tandab (CD3/CD19) resulted in enhanced cytotoxicity. These results are in accordance with our earlier report on diabody of antiCD19/antiCD3 [23].

### MSCs can be successfully transduced to release Tandab (CD3/CD19)

**Fig. 2:**
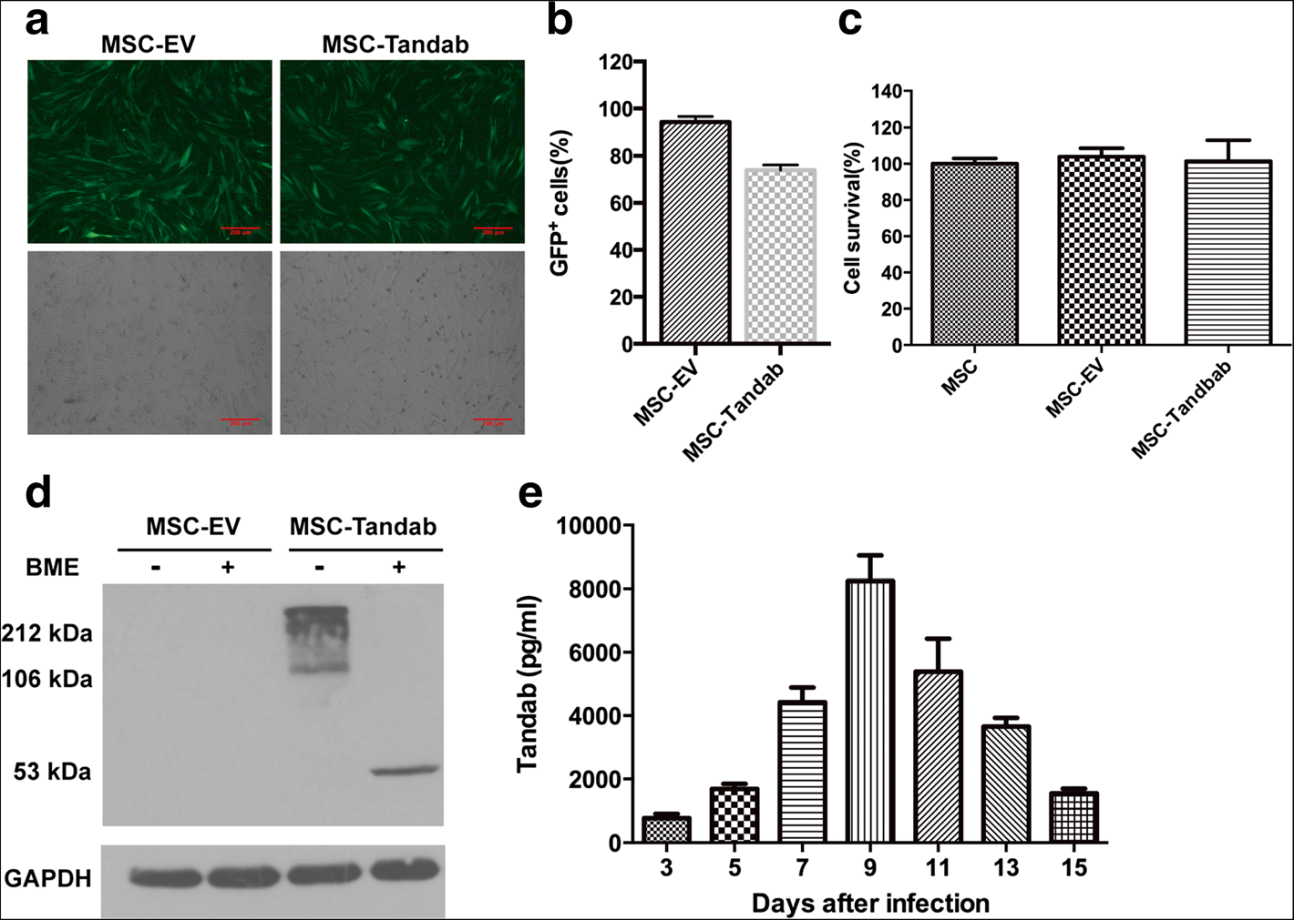
Constitutive expression of Tandab (CD3/CD19) in MSCs. MSCs were transduced with lentivirus coding Tandab (CD3/CD19) at 8 MOI for 12 h. Then, supernatants were removed, and fresh medium culture was added. **a** The representative images depicted the infection efficiency of MSCs with lentivirus. Forty-eight hours after infection, MSCs carrying copGFP were observed under fluorescent field (*upper panel*) and bright field (*lower panel*), *scale bar* = μm. MSC-Tandab, MSCs transduced with lentivirus coding Tandab (CD3/CD19); MSC-EV, MSCs transduced with empty lentivirus. **b** FACS analysis of percentages of copGFP-positive MSCs. **c** Cell survival of MSCs transduced with or without lentivirus were detected by the MTT assay. **d** Western blot analysis was employed to determine the protein expression of Tandab (CD3/CD19) in MSCs with anti-His tag antibodies after 5 days of transduction. GAPDH, served as a loading control; BME, β-mercaptoethanol. **e** Transduced MSCs secreted Tandab (CD3/CD19) constantly. MSC-Tandab and MSC-EV were cultured in a 24-well plate (4 × 10^4^/well). And the level of Tandab (CD3/CD19) released into culture was measured by ELISA in the indicated time. Data shown are the mean ± SD of the three repeated experiments

MSCs were isolated from human umbilical cord and identified according to previous reports [24, 25]. Then, MSCs were transduced with lentivirus encoding Tandab (CD3/CD19) with copGFP (MSC-Tandab) or alone copGFP (MSC-EV). The morphology of these cells and their transduction efficiency (indicated by copGFP) are shown in Fig. 2a. The transduction efficiency was also assessed by FACS analysis, which indicated that more than 73% of the MSCs were successfully transduced (Fig. 2b). And no significant alterations in cell survival were observed among MSC-Tandab, MSC-EV, and wide-type MSCs (Fig. 2c). Western blot results demonstrated that Tandab (CD3/CD19) was highly expressed in MSC-Tandab but not in MSC-EV (Fig. 2d). Additionally, the Tandab (CD3/CD19) released in the supernatants in culture of MSC-Tandab was detected by ELISA. The level of secreted Tandab (CD3/CD19) achieved a peak at day 9 (8247.2 ± 796.7 pg/mL) and was detectable even at day 15 after transduction (Fig. 2e). And there was no difference on surface markers between MSCs and transduced MSCs (Additional file 2: Fig. S1A). In addition, gene-modified MSCs maintained their ability of tri-lineages differentiation (Additional file 2: Fig. S1B and C).

### Homing property of MSCs to B cell lymphoma in vitro and in vivo

**Fig. 3:**
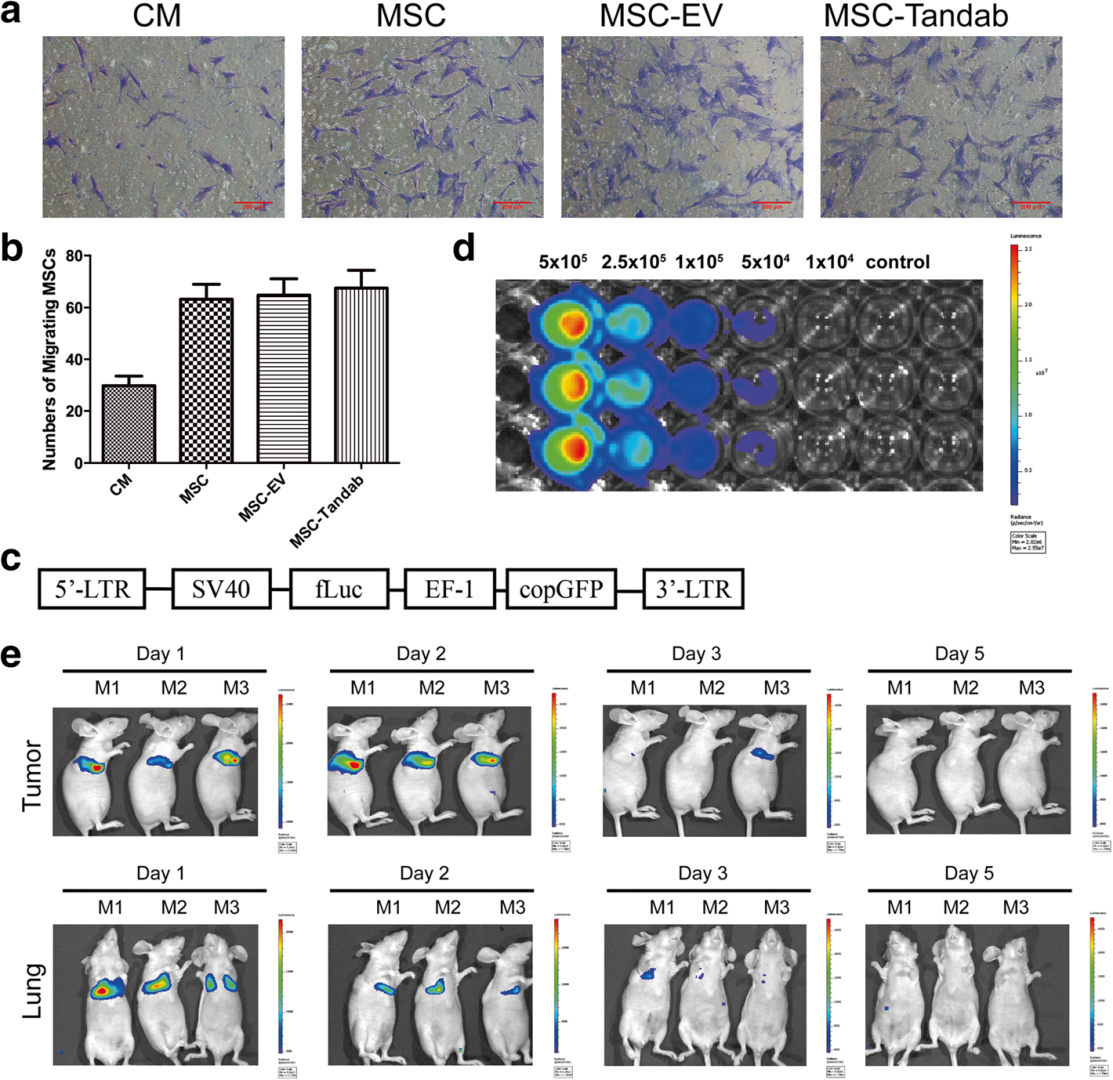
Migration capacity of MSCs to B cell lymphoma in vitro and in vivo. **a** Representative photographs showed the migrated MSCs stained with crystal violet in vitro migration assays. Culture medium (*CM*), served as a negative control. *Scale bar* = 200 μm. **b** The numbers of migrated MSCs in three independent assays were expressed as mean ± SD. **c** Schematic representation of lentiviral expression vector for firefly luciferase (*Luc*). **d** MSC-Luc expressed Luc constitutively in vitro. **e** Tropism of MSCs to tumor site. MSC-Luc was intravenously injected into tumor-bearing mice. The mice were anesthetized in the indicated time and received intraperitoneal injection of d-luciferin at a dose of 150 μg of d-luciferin per gram of body weight. Ten minutes later, the BLI for luciferase activity was detected by Xenogen in vivo imaging system. *M1*, *M2*, *M3*: represented three mice

MSCs and gene-modified MSCs were previously proved to have a homing predisposition to tumor cells in vitro and to the tumor site in models of hepatocarcinoma with HepG2 cells or lymphoma with BJAB cells [12, 13, 25]. To investigate the migration capacity of transduced MSCs to another B cell lymphoma cell line (Raji cells) in this study, migration assays in vitro using transwell plates were performed. Culture medium (CM) without Raji cells was served as a negative control of chemotaxis, and unmodified MSCs were used as a positive control of migrating cells. We confirmed that the transduced MSCs migrated towards Raji cultures in a similar pattern as unmodified MSCs (Fig. 3a, b). These results indicated that human B cell lymphoma Raji cells were able to stimulate the migration of MSCs and the migration capacity of MSCs was not affected by infection of lentivirus.

In order to verify the homing ability of MSCs in vivo, we designed a lentiviral vector (pLeniR-Luc) harboring a firefly luciferase reporter gene (Fig. 3c). MSCs labeled with luciferase (MSC-Luc) were analyzed at first ex vivo using bioluminescence imaging (BLI) system (Fig. 3d), which suggested that luciferase reporter gene could be used to quantify transplanted MSCs in small living animals. Then, MSC-Luc (1 × 10^6^ cells per mouse) was injected intravenously into BALB/c nude mice with established subcutaneously tumor with Raji cells. BLI revealed that MSCs migrated and selectively accumulated at the tumor site at 24 h after injection although part of MSCs was hijacked in the lungs, in which the signal decreased as time went on (Fig. 3e). The strongest signal in the tumor site was observed on day 2, and it was out of detectable on day 5 (Fig. 3e). During the monitoring process, MSCs were not traced in other tissues.

### MSC-Tandab exhibits cytotoxicity against CD19-positive Raji cells

**Fig. 4:**
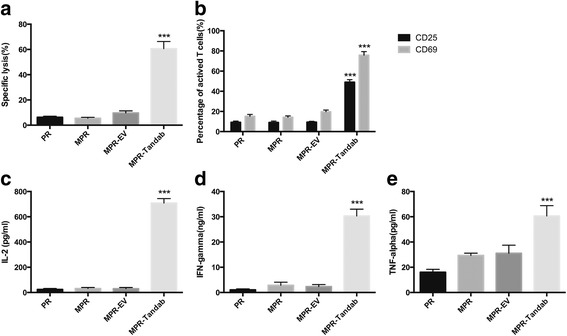
Cytotoxicity of T cells to Raji cells mediated by Tandab (CD3/CD19) secreted from MSCs. In order to evaluate the function of MSCs-secreting Tandab (CD3/CD19), a co-culture system using transwell plates with 0.4-μm-pore membrane was established. MSCs were plated into 6-well plates with a density of 1 × 10^5^ cells per well after transduced with lentivirus. Seventy-two hours later, Raji cells were labeled with calcein-AM (5 μM). Then, PBMCs and labeled Raji cells (E:T=10:1) were added to the equilibrated inserts. After co-cultured for 24 h, cells in the *inserts* were harvested to be detected by FACS. **a** The specific lysis of Raji cells. **b** Activation surface markers CD69 and CD25 of T cells. **c**–**e** Cytokines including IL-2, IFN-*γ*, and TNF-*α* in the supernatant were measured using ELISA kits. *PR*, PBMC + Raji; *MPR*, MSC + PBMC+Raji; *MPR-EV*, MSC-EV + PBMC+Raji; *MPR-Tandab*, MSC-Tandab + PBMC + Raji. ***P< 0.001 compared with PR group. Data shown are the mean ± SD of the three repeated experiments

To confirm the bioactivity of secreted Tandab (CD3/CD19) from MSC-Tandab, the co-culture system using transwell plates with 0.4-μm-pore membrane (cells could not pass through) was designed. MSC-secreting Tandab (CD3/CD19), concentration of which could reach (0.1516 ± 0.0283) pmol/mL, in the lower chamber could pass through the membrane followed by triggering reactions between PBMCs and Raji cells (E:T = 10:1). After incubation for 24 h, obviously lysis (60.6 ± 5.7%) of Raji cells was detected by flow cytometry (Fig. 4a). The expression levels of the early T cell activation marker CD69 and of the late activation marker CD25 on CD3-posivitve cells were also assessed (Fig. 4b). Since the increase of cytokines concentration is the response of T cells activation and cytotoxicity, classic cytokines including IL-2, IFN-γ, and TNF-α were measured as an example to evaluate the activation efficacy of T cells in the co-culture system. The concentrations of IL-2, IFN-γ, and TNF-α were (708.27 ± 36.16) pg/mL, (30.31 ± 2.69) ng/mL, and (60.66 ± 8.21) pg/mL in the supernatant, respectively, all of which were significantly higher than that of the control groups (*P* < 0.001) (Fig. 4c–e). These data suggest that MSC-Tandab can trigger cytotoxicity to CD19-positive tumor cells in the presence of effector cells.

### The cytotoxicity induced by MSC-secreting Tandab (CD3/CD19) can be enhanced by IDO pathway inhibitor D-1MT

**Fig. 5:**
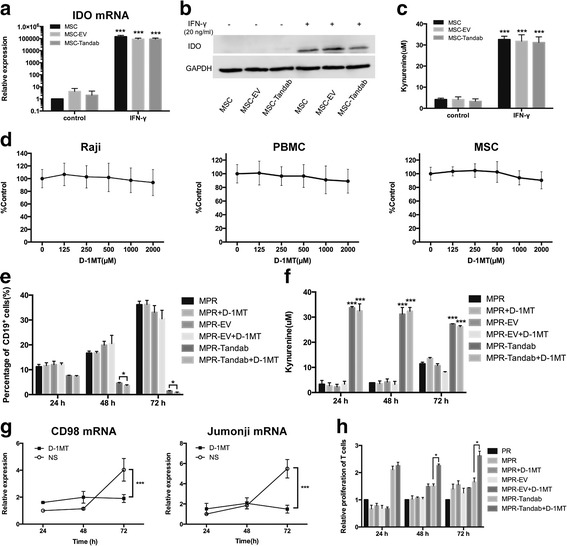
IDO pathway inhibitor D-1MT enhances the cytotoxicity induced by MSC-secreting Tandab (CD3/CD19) in vitro. **a** IDO mRNA expression in MSCs stimulated with IFN-γ. MSCs were cultured in the absence or presence of exogenous IFN-γ (20 ng/mL). Cells were harvested after 2 days in culture, total mRNA was extracted, and IDO message was assayed by real-time PCR. **b** IDO protein was determined by Western blot analysis. MSCs were harvested after cultured for 48 h, as in **a**, and total cell lysates were assayed for IDO protein. Results are representative of three independent experiments. **c** To determine enzyme activity of IDO, MSCs were cultured with or without IFN-γ (20 ng/mL) stimulation for 48 h. IDO enzyme activity was evaluated by spectrophotometric detection of the tryptophan metabolite, kynurenine, a product of IDO catabolism. **d** Influence of D-1MT on the proliferation of cells (Raji, PBMCs and MSCs). Cells were seeded in 96-well plates and treated with different concentrations of D-1MT (0–2000 μM) for 72 h. Cell proliferation was determined by MTT assay, and the *y* axis represents cell proliferation as a percent of the control. **e** D-1MT promotes the specific lysis of Raji cells induced by MSC-secreting Tandab (CD3/CD19). MSCs, Raji cells, and PBMCs were co-cultured as mentioned above with or without D-1MT (1 mM) for 24, 48, and 72 h. The residual Raji cells were detected by FACS with FITC-conjugated anti-CD19 antibodies. *D-1MT*, d-1-methyl-tryptophan. **f** Determination of kynurenine in the supernatant from the co-culture system. **g** Analysis of anergy-associated genes expression of CD98 (*left panel*) and Jumonji (*right panel*). The cells in the *inserts* were harvested in the indicated time. Total mRNA was extracted, and the messages of CD98 and Jumonji were assayed by real-time PCR. *NS*, normal saline. **h** Relative proliferation of T cells. The cells were harvested in the indicated time. Proliferation of T cells was detected using BrdU flow kit. The proliferation of T cells in PR group (without MSCs) was served as a reference. *P < 0.05 compared with the control group; ***P < 0.001 compared with the control group. Data shown are the mean ± SD of the three repeated experiments

Although we have demonstrated that CD19-positive tumor cells could be killed efficiently by T cells in the co-culture system, the presence of MSCs should be in consideration because of their great immune-modulating capacity. Some earlier reports have indicated that IDO plays a pivotal role in MSC-mediated immunosuppression [17, 31, 32] because IDO expression by human MSCs occurs only after exposure to inflammatory cytokines such as IFN-γ [31, 33], which produced massively by T cells during the co-culture assays. For this reason, an IDO pathway inhibitor D-1MT was employed in our study to overcome the immunosuppression induced by MSCs. We firstly verified the inducible expression of IDO in MSCs with stimulation of exogenous IFN-γ. After stimulation with IFN-γ (20 ng/mL) for 48 h, the expression of IDO were detected at the mRNA level and protein level (Fig. 5a, b). Furthermore, the enzymatic activity of IDO (indicated by concentration of kynurenine, a metabolism of tryptophan) was measured (Fig. 5c). We next determined whether D-1MT had any cytotoxicity on MSCs, Raji cells, or PBMCs. Results from the cell proliferation assays suggested that no obvious influences were caused by D-1MT on those cells (Fig. 5d). Based on these results, we then performed a co-culture killing experiment again in the same conditions described above in the absence or presence of D-1MT (1 mM) for 24, 48, and 72 h. As shown in Fig. 5e, the number of residual Raji cells decreased significantly at 48 and 72 h (*P* < 0.05). However, there was no significant difference at the first 24 h.

To further investigate the mechanism of D-1MT exerted in the increasing cytotoxicity, we firstly pointed to the level of kynurenine which had no changes from 24 to 72 h (Fig. 5f). Previously, reports show that D-1MT can act as a mimetic of tryptophan in the sufficiency pathway, thereby functionally reversing the effects of IDO on formation of T cell anergy controlled by PKC-θ [22]. We therefore examined the effect of D-1MT on the expression of anergy-associated genes in T cells, including CD98 and Jumonji [34]. Both of the expression of CD98 and Jumonji in T cells from the co-culture system were up-regulated at 72 h, but this effect was reversed efficiently by D-1MT (Fig. 5g). Interestingly, the proliferation of T cells in the co-culture system was also partly restored by D-1MT at 48 and 72 h, respectively (Fig. 5h).

### Antitumor potential of MSC-Tandab in combination with D-1MT against Raji xenograft tumors

**Fig. 6:**
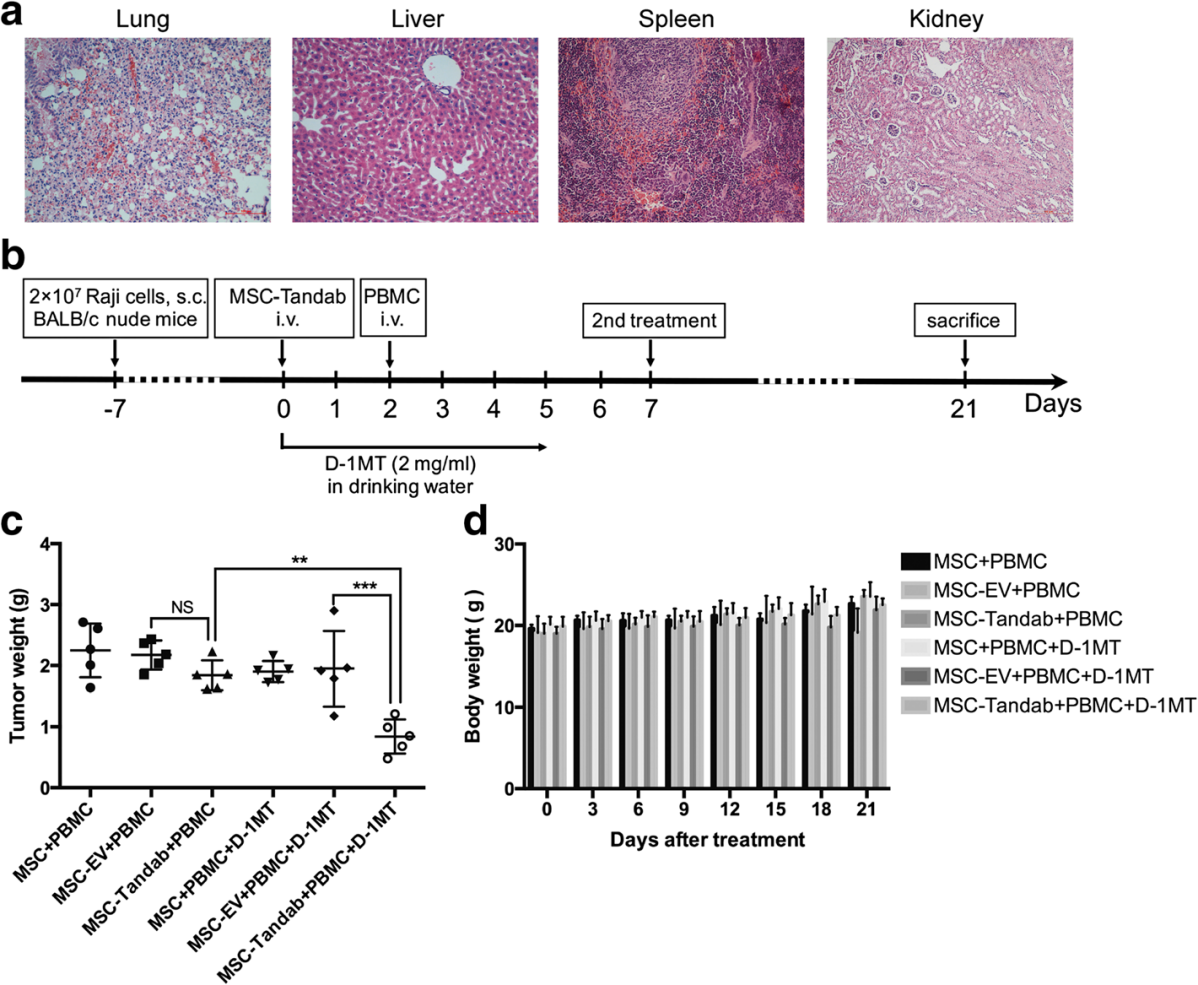
Tumor suppressing effects of MSC-Tandab in combination with D-1MT against B cell lymphoma in mice. **a** Histological analysis in organs from mice. On day 7 after treatment, mice organs including the *lung*, *liver*, *spleen*, and *kidney* were dissociated for fixation and HE staining. *Scale bar* = 100 μm. **b** Experimental protocol of the tumor therapy. BALB/c nude mice were injected subcutaneously with Raji cells (2 × 10^7^ per mouse) into the right flank. Seven days later (day 0), MSCs (1 × 10^6^ per mouse) were injected intravenously into the xenograft Raji tumor-bearing mice. Mice received intravenous injection of PBMCs (5 × 10^6^ per mouse) on day 2. And mice were treated with or without D-1MT (2 mg/mL in the drinking water) along the treatment. The second treatment was received on day 7. All mice were sacrificed on day 21. **c** The tumor weights of different groups were measured in the end of treatment. **d** Changes in the body weight of Raji lymphoma xenograft models during the treatment. *NS*, no significance; **P<0.01; ***P<0.001

We further investigated the antitumor potential of MSCs engineered with Tandab (CD3/CD19) in combination with D-1MT in BALB/c nude mice subcutaneously implanted with Raji cells. Firstly, to ascertain the safety of this therapeutic schedule, tumor-bearing mice were treated with MSC-Tandab, PBMCs, and D-1MT. Results from pathological reports indicated that this treatment did not cause organ damages, such as the lung, liver, spleen, and kidney (Fig. 6a). Then, we begun the formal therapeutic experiments. When the tumor size reached 100–200 mm^3^, the tumor-bearing mice were grouped and treatments were started according to the scheme shown in Fig. 6b. The animals were sacrificed on day 21, and tumor tissues were dissected and weighed for analysis. Interestingly, compared with the mice in the control group (treated with MSC-EV + PBMC), the mice treated with MSC-Tandab and PBMCs exhibited no evident tumor regression (Fig. 6c). However, the tumor weight of the mice treated with MSC-Tandab and PBMCs combined with D-1MT decreased 61.2% (Fig. 6c). In addition, no obvious changes in the body weight of the mice were observed during the treatment (Fig. 6d).

## Discussion

Application of MSCs to serve as vehicles for delivering TandAb to tumor has not been reported so far. In this study, we investigated the therapeutic effects of gene-modified MSCs with Tandab (CD3/CD19) for B cell lymphoma. The hypothesis is that engineered MSCs which injected intravenously into the tumor-bearing mice would specifically migrate to tumor site and secrete Tandab (CD3/CD19) which recruits T cells to exhibit a potent antitumor immunity in combination with an IDO pathway inhibitor D-1MT. And results presented here support the feasibility of this strategy.

Currently, two approaches have been developed to harness T cells for killing tumor cells. The first approach utilizes gene-modified T cells with an engineered chimeric antigen receptor (CAR) combining a desired antigen-recognition fragment of a monoclonal antibody against T cell activation domains from the T cell receptor complex, such as the ζ chain, with co-stimulatory molecules, from CD28, 4-1BB, or OX40 [35]. The other approach depends on T cell recruitment via bispecific antibodies (bsAbs) [36] which bind one arm to T cell activation domain and bind the other arm to a tumor-associated antigen on the target cell. We previously constructed an anti-CD3 × anti-CD19 bispecific antibody, which could efficiently redirect T cells to lysis both B cell lymphoma cell lines and patient-derived B-ALL cells [23, 37]. In this study, we successfully constructed a new tandem diabody with two binding sites for CD3 and two for CD19 using the DNA sequences from our anti-CD3 × anti-CD19 bispecific antibody. The relative amount of tandem diabodies proved to be dependent on the length of the linker in the middle of the chain [7]. It was reported that the middle linker should not be too short or too long to force the four domains to interact with complementary ones of another molecule with the formation of an eight-domain Tandab [7]. We therefore chose a linker with 15 amino acid residues in our construct. In addition, our earlier data have suggested that the introduced disulfide covalent bond between V_H_3 and V_L_3 at rational positions could enhance the stability of fusion proteins [23, 38]. Since the two peptides of TandAb are held together by non-covalent associations of the corresponding V_H_ and V_L_ domains, an extra disulfide bond can prevent the peptide diffusing away from the other one. Results from Western blot analysis suggested that nearly no monomers were detectable in the non-reducing condition. However, we also observed a band in 212 kDa in the non-reducing condition (Figs. 1c, d and 2d), which should be dimers of Tandab (CD3/CD19) with intermolecular forces. And the amount of them decreased after purification (Fig. 1d). Furthermore, data from cell-binding assays and cytotoxicity assays in vitro indicated that our Tandab (CD3/CD19) could bind to both of CD3-positive cells and CD19-positive cells and induce the specific lysis of CD19-positive cells.

MSCs hold great promise for clinical applications in the treatment of various diseases owing to their multi-lineage differentiation potential and immunosuppressive properties. Furthermore, the tumor tropism property of MSCs has led to the utilization of them as attractive delivery vehicles for a spectrum of antitumor agents against cancers [39–43]. Several previous investigations reported by our laboratory also support feasibility of this strategy [12, 13, 25, 44]. In the present study, we engineered the human umbilical cord-derived MSCs through transduction by lentivirus to secrete Tandab (CD3/CD19). We demonstrated that the MSC-Tandab could maintain their properties after lentiviral infection, including their surface markers, tri-lineage differentiation, proliferation, and the capacity to migrate towards tumor cells in vitro. Additionally, the tropism towards tumor-bearing mice with Raji cells was confirmed by in vivo imaging system with luciferase-labeled MSCs (Fig. 3e). These findings indicate that UC-MSCs can be used as ideal vehicles to deliver Tandab (CD3/CD19) to tumor.

Furthermore, to mimic the therapeutic process with MSC-Tandab in vivo, we designed a co-culture system using transwell plates mentioned above, in which Tandab (CD3/CD19) secreted from MSC-Tandab could migrate to the upper insert to trigger cytotoxicity of T cells against CD19-positive cells. Results from the specific lysis of Raji cells and released cytokines confirmed our initial hypothesis. However, it has been reported that various factors are believed to be involved in the mechanisms of MSC-mediated immunosuppression, including inducible nitric oxide synthase (iNOS), IDO, tumor necrosis factor-inducible gene 6 (TSG6), CC-chemokine ligand 2 (CCL2), IL-10, and prostaglandin E2 (PGE2) [45]. Although the immune-regulatory effects of MSCs do not occur spontaneously, it should be taken into account in this study because of the released cytokines, such as IFN-γ. The expression of IDO in MSCs induced by IFN-γ might contribute to the immunosuppressive microenvironment owing to tryptophan depletion and accumulation of its metabolites. Additionally, MSC-Tandab shows basal increased levels of kynurenine (Fig. 5f), which is associated to an enhanced immunosuppression ability. This aspect indicates that we should pay attention to genetic modification of MSCs since they could be responsible of an augmented pro-tumoral effect. Therefore, we employed D-1MT, an IDO pathway inhibitor which differed from direct enzymatic inhibitors of IDO, to overcome the inhibition effect of IDO. Data from the co-culture killing experiments showed that D-1MT could increase the ratio of lytic cells at a certain degree. No significant changes on the level of kynurenine were observed, which suggested that D-1MT could not inhibit the enzymatic activity of IDO directly. Then, we confirmed that the expression of the T cell anergy-associated genes CD98 and Jumonji were decreased in the presence of D-1MT. And the proliferation capacity of T cells was also increased by D-1MT. Furthermore, the antitumor growth effect of MSC-Tandab in vivo was enhanced significantly in combination with D-MT. Although IDO is known to be overexpressed in several human cancers, including prostate, breast, brain, and hematologic malignancies [46], it is not detectable in Raji cells even with the stimulation of IFN-γ [47]. While a study about IDO expression by circulating leukemic cells from 5 B-CLL patients showed that IDO could not be detected in the PBMCs of any of them, but in four of the five, IDO was dramatically up-regulated when cells were cultured with IFN-γ for 24 h [47]. This may be one of the reasons for drug resistance during the treatment. Thus, our treatment with MSC-Tandab in combination with IDO pathway inhibitor D-1MT indicates a new treatment strategy for refractory and relapse B cell malignancies.

## Conclusions

We have demonstrated in this study that human UC-MSCs can be acted as cell-based delivery vehicles for the treatment of B cell lymphoma. Tandab (CD3/CD19) secreted from UC-MSCs effectively redirected T cells to inhibit the growth of Raji lymphoma in mouse models in combination with D-1MT. Our findings indicate that the use of UC-MSCs as vehicles for engineered-antibodies combined with immunoregulatory agents represents a potential clinical application of gene therapy for cancers.

## Additional files

Additional file 1: Supplementary methods and materials. (DOCX 100 kb)
Additional file 2: Figure S1. Identification of MSCs and transduced MSCs. A. Phenotypic analysis of MSCs and transduced MSCs. Flow cytometric analysis showed that MSCs, MSC-EV, and MSC-Tandab expressed CD73, CD90, and CD105 but not CD14, CD19, CD34, CD45, and HLA-DR. B. Representative images showing the in vitro differentiation of MSCs and transduced MSCs into adipogenic, osteogenic, and chondrogenic lineages, respectively. Scale bar = 200 μm. C. Relative quantification of gene expression after tri-lineage differentiation in MSCs and transduced MSCs. The mRNA levels were normalized using the expression of the reference gene (GAPDH) and compared with the MSCs group. Data shown are the mean ± SD of three repeated experiments. (TIF 9594 kb)

## Abbreviations

AHR: Aryl hydrocarbon receptor
B-ALL: B-precursor acute lymphoblastic leukemia
BiTE: Bispecific T cell engager
BLI: Bioluminescent imaging
B-NHL: B cell non-Hodgkin’s lymphoma
BrdU: Bromodeoxyuridine
bsAbs: Bispecific antibodies
CAR: Chimeric antigen receptor
CM: Culture medium
D-1MT: d-1-methly-tryptophan
ELISA: Enzyme-linked immune sorbent assay
EV: Empty vector
FDA: Food and Drug Administration
GAS: γ-activating sequences
IDO: Indoleamine 2,3-dioxygenase
ISRE: Interferon stimulatory response elements
Luc: Luciferase
MOI: Multiplicity of infection
MRD: Minimal residue diseases
MSCs: Mesenchymal stromal cells
PBMCs: Peripheral blood mononuclear cells
PBS: Phosphate-buffered saline solution
PCR: Polymerase chain reaction
scFv: Single-chain variable fragment
TandAb: Tandem diabody
UC: Umbilical cord
WJ: Wharton’s jelly
