# Supplementary material for: Mesenchymal stromal cells as vehicles of tetravalent bispecific Tandab (CD3/CD19) for the treatment of B cell lymphoma combined with IDO pathway inhibitor d-1-methyl-tryptophan
Source: J Hematol Oncol. 2017 Feb 23;10:56. doi: 10.1186/s13045-017-0397-z (PMC5322661; doi:10.1186/s13045-017-0397-z)
Supplement: Additional file 1: — Supplementary methods and materials. (DOCX 100 kb) [file 13045_2017_397_MOESM1_ESM.docx]

**Supplementary Methods and Materials**

PBMCs isolation

Human peripheral blood mononuclear cells (PBMCs) were isolated from peripheral blood of healthy volunteers by Ficoll-Hypaque density-gradient centrifugation (Institute of Hematology & Blood Diseases Hospital, Chinese Academy of Medical Science & Peking Union Medical College, Tianjin, China). Cells of the interphase were washed twice with phosphate-buffered saline solution (PBS) followed by incubation in erythrocyte lysis buffer (150 mM NH4Cl, 10 mM KHCO3, 100 $\mu$M EDTA) for 10 min at room temperature. Then cells were centrifuged for 5 min at 600$\times$ *g*. The supernatant containing the lysed erythrocytes was discarded and the cell pellet was resuspended in PBS. Platelets were removed in additional centrifugation step for 15 min at 100$\times$*g*. Then cells were cultured in RPMI-1640 medium (Invitrogen, USA) supplemented by 10% FBS and 50 IU/mL interleukin 2 (IL-2) for 48 h.

Construction of lentiviral expression vectors

The Tandab (CD3/CD19) sequence with a domain arrangement V_L_3-V_H_19-V_L_19-V_H_3 was generated by fusing V_L_3-V_H_19 and V_L_19-V_H_3 via a flexible peptide linker (G4S)_3_ by overlap PCR. Then the murine kappa light-chain leader peptide was genetically linked to the N-terminus of the fusion fragment Tandab (CD3/CD19). The His-tag (C-terminus) and the corresponding restriction enzymes *Nco*$= 1 \backslash* ROMAN I$ (N-terminus) and *Xba*$= 1 \backslash* ROMAN I$ (C-terminus) were introduced. Then the cDNAs of Tandab (CD3/CD19) were inserted into pGL3-control vector (Promega, USA) for replacement of luciferase genes, in which the elements of SV40 promoter, enhancer, and poly A were employed. Finally, the expression cassette of Tandab (CD3/CD19) was cloned into the lentiviral expression vector pCDH (Cat. No. CD511B-1, System Biosciences, SBI, USA) with restriction enzymes *Nhe*$= 1 \backslash* ROMAN I$ (N-terminus) and *BamH*$= 1 \backslash* ROMAN I$ (C-terminus). The construction was named pLentiR-Tandab (CD3/CD19), and was confirmed by DNA sequence analysis (Invitrogen, USA). Plasmid pLentiR-EV (empty vector) was served as vector control. All lentiviral packaging plasmids (Cat. No. LV100A-1, SBI, USA) and the lentiviral expression vector pCDH (Cat. No. CD511B-1, SBI, USA) were kindly provided by professor Xiaotong Ma (PUMC).

Cell binding assay

1$\times$10^6^ cells were incubated with purified Tandab (CD3/CD19) at 5 pmol/mL in a volume of 200 $\mu$L for 1 h at 4 $℃$. After washed three times with cold PBS, the cells were incubated with 100$\mu$L Alexa Fluor 488 labeled anti-His tag antibody (MBL, Japan) at 0.5 $\mu$g/mL for an additional 30 min. Then the stained cells were washed and analyzed by flow cytometry. In the competitive binding assay, 1$\times$10^6^ cells were firstly incubated with 200 $\mu$L purified Tandab (CD3/CD19) at 5 pmol/mL for 1 h at 4 $℃$, then incubated with FITC-conjugated HIT19a or PE-conjugated HIT3a (Institute of Hematology & Blood Diseases Hospital, Chinese Academy of Medical Science & Peking Union Medical College, Tianjin, China) for 30 min at 4 $℃$. After being washed three times and resuspended with PBS, cells were detected by flow cytometry.

Cytotoxicity assay

CytoTox96^®^ Non-Radioactive Cytotoxicity Assay (also known as LDH release assay; Promega, USA) was used to assess the specific lysis of target cells according to the manufacture’s protocol. Briefly, quadruplicates of 1$\times$10^5^ target cells and PBMCs at different effector to target (E:T) cell ratios ranging from 20:1 to 1:1 were added to 96-well culture plates in a final volume of 100 $\mu$L with additional 100 $\mu$L of Tandab (CD3/CD19) (8 pmol/mL). At the same time, target cells and PBMCs were seeded to additional 96-well culture plates with different concentrations of Tandab (CD3/CD19) at the same E:T ratio (20:1) in a final volume of 200 $\mu$L. Three repeated wells were designed. After 8 h, supernatants were transferred to a fresh 96-well flat clear bottom plate for detection. The OD value was detected at 490 nm with a synergy H4 microplate reader (BioTek, USA) and cytotoxicity was calculated according to the formula: $\% Cytotoxicity=\frac{Experimental-Effector Spontaneous-Target Spontaneous}{Target Maximum-Target Spontaneous} \times100$. In order to verify the specific cytotoxicity of Tandab (CD3/CD19), CD19-negative cells K562 were used as control target cells in above experiments.

Production of lentivirus

The lentiviral particles were produced in 293T cells according to the SBI’s protocols. Briefly, 8 $\times$10^6^ 293T cells were seeded in a fresh 10-cm dish (Corning, USA) the day before transfection. Mixing 10 $\mu$g of the pPACK Packing Plasmid Mix with 2 $\mu$g of lentivector expression construct and diluted into 500 $\mu$L of OPTI-MEM (Gibco, USA); diluting 30 $\mu$L of Lipofectamine 2000 Reagent with 500 $\mu$L of OPTI-MEM at room temperature for 5 min. Diluted Lipofectamine 2000 was added into diluted plasmids. The complex was mixed gently by inversion, and incubated at room temperature for 20 min. Then the complex was added into the dish of 293T culture. Supernatants were harvested at 48 h post-transfection. The lentivirus-containing supernatants were centrifuged at 500 $\times$ *g* for 10 min at 4 $℃$, filtered through a 0.45 $\mu$m pore size filter (Millipore, USA), and used to infect MSCs immediately or stored at -80 $℃$.

Transduction of MSCs and viability of transduced MSCs

MSCs were plated at a density of 8000 cells/cm^2^ in a T-75 plastic culture flask and incubated overnight at 37 $℃$. On next day, culture medium was removed and 8 mL of fresh medium containing lentivirus at a multiplicity of infection (MOI) of 8 pfu per cell with 8 $\mu$g/mL of polybrene (Sigma-Aldrich, USA). The medium was removed after 8 h, and fresh DF-12 medium containing 10% FBS was added. MSCs were incubated for another 48 h and observed under a fluorescence microscope. And the efficiency of infection was determined by flow cytometry. Western blot analysis was applied to detect the expression of desired protein 5 days after transduction. Furthermore, transduced MSCs were seeded into 24-well culture plate at a density of 4$\times$10^4^ cells per well. The concentrations of MSCs-secreting Tandab (CD3/CD19) in the supernatants were measured by His-tag ELISA detection kit (GenScript, USA) at the indicated time points.

To verify the influence induced by infection of lentivirus on MSCs, viability of transduced MSCs was assessed. MSCs infected by lentiR-EV or lentiR-Tandab (CD3/CD19) were named MSC-EV and MSC-Tandab, respectively. And wide-type MSCs were served as control. These three kinds of MSCs were plated into 96-well culture plates at a density of 3000 cells per well. The cell viability was detected by MTT assay (Sigma-Aldrich, USA) after 72 h. The absorbance of each well was measured at 570 nm with a synergy H4 microplate reader (BioTek, USA).

In vitro and in vivo MSC migration assay

The migration of transduced MSCs *in vitro* was determined using 8 $\mu$m pore membrane inserts with 6.5 mm diameter (BD Falcon, USA). 24 h after infection, 4 $\times$10^4^ MSCs were seeded in the top chamber in 400 $\mu$L of serum-free medium. The previous day, Raji cells were plated at a density of 6$\times$10^5^ cells per well in the lower chamber in RPMI-1640 medium containing 10% FBS. Cell-free medium was served as negative control. After 12 h of incubation at 37 $℃$, cells that had not migrated from the upper side of the membrane were scraped off with a cotton swab, and cells in the lower side of the membrane were stained with 0.1% crystal violet at 37 $℃$ for 45 min. The number of cells that had migrated to the lower side of the membrane was quantified in five randomly selected high-power ($\times$200) microscope fields. Experiments were performed in triplicate.

For *in vivo* migration assay, we developed a lentiviral expression vector containing a firefly luciferase reporter gene (pLentiR-Luc) to label MSCs. BALB/c nude mice (female, 6 weeks old, Peking Union Medical College, PUMC, China) were challenged with Raji cells in 200 $\mu$L of PBS at the right flank of the animals. Seven days later when tumors reached 100-200 mm^3^ in size, 1$\times$10^6^ MSCs labeled with luciferase (MSC-Luc) were injected intravenously. Bioluminescence imaging (BLI) was performed using IVIS-Xenogen 100 system (Caliper Lifesciences, USA) at indicated time.

T cell proliferation assay

Proliferation of T cells were detected using BrdU Flow Kit (BD Bioscences) according to the manufacture’s protocol. Briefly, BrdU (10 $\mu$M) was added to cells in the upper-chamber for incubation for 1 hour. Then, cells were harvested and washed with PBS followed by staining cell-surface marker with FITC-labeled anti-CD3 antibodies. After washed with staining buffer, cells were fixed and permeabilized with BD Cytofix/Cytoperm Buffer. Next, cells were incubated with BD Cytoperm Permeabilization Buffer Plus for 10 min on ice followed by re-fixation. Then, cells were treated with DNase (1 mg/mL) for 1 hour at 37$℃$ to expose incorporated BrdU. Finally, cells were stained with APC-labeled anti-BrdU antibodies for 20 min before analysis on a flow cytometer. And the proliferation of T cells in PR (PBMC+Raji) group without MSCs was serve as control.

Detection of kynurenine

Two hundred microliters of cell culture were mixed with 100 $\mu$L of 30% trichloroacetic acid and incubated at 50 $℃$ for 30 min. After centrifugation (12 000 $\times$g for 1 min), 125 $\mu$L supernatant was mixed with an equal volume of Ehrlich’s reagent (100 mg p-simerthylbenzaldehyde in 5 mL glacial acetic acid) in a 96-well plate. The OD value of samples was read with a synergy H4 microplate reader (BioTek, USA) at 490 nm. The concentration of kynurenine in samples was calculated according to the standard curve established with commercial-obtained kynurenine (Sigma-Aldrich, USA).

Cell viability assay

Raji cells (2$\times$10^4^ cells per well), PBMCs (1$\times$10^5^ cells per well), and MSCs (5000 cells per well) were cultured with various concentrations of D-1MT (0-2 mM) in 96-well flat-bottomed plates for 72 h. Then an MTT cell viability assay was performed daily according to the manufacturer’s instruction. The cells treated with PBS were served as a control. All experiments were performed in triplicate. The results were presented as a percentage of the control, which was considered to be 100%.

Western blot analysis

Cells were harvested and washed three times with PBS, and resuspended in 100 $\mu$L RIPA lysis buffer (Beyotime Biotechnology, China). Protein concentrations were determined using BCA assay (Pierce, USA). Aliquots of protein lysates were separated on a 12% SDS-polyacrylamide gels and transferred onto a nitrocellulose membrane, which was blocked with 5% blotting grade milk (Bio-Rad, USA) in PBST (0.1% Tween-20 in PBS). The membrane was then probed with the indicated primary antibodies to His-tag, human IDO or GAPDH followed by corresponding second antibodies conjugated with horseradish peroxidase, and then detected using a chemiluminescence assay (Millipore, USA). Membranes were exposed in Image Quant LAS-4000 (GE Healthcare, Sweden) to visualize the bands.
