# Supplementary figures and images for: Mesenchymal stromal cells as vehicles of tetravalent bispecific Tandab (CD3/CD19) for the treatment of B cell lymphoma combined with IDO pathway inhibitor d-1-methyl-tryptophan
Source: J Hematol Oncol. 2017 Feb 23;10:56. doi: 10.1186/s13045-017-0397-z (PMC5322661; doi:10.1186/s13045-017-0397-z)

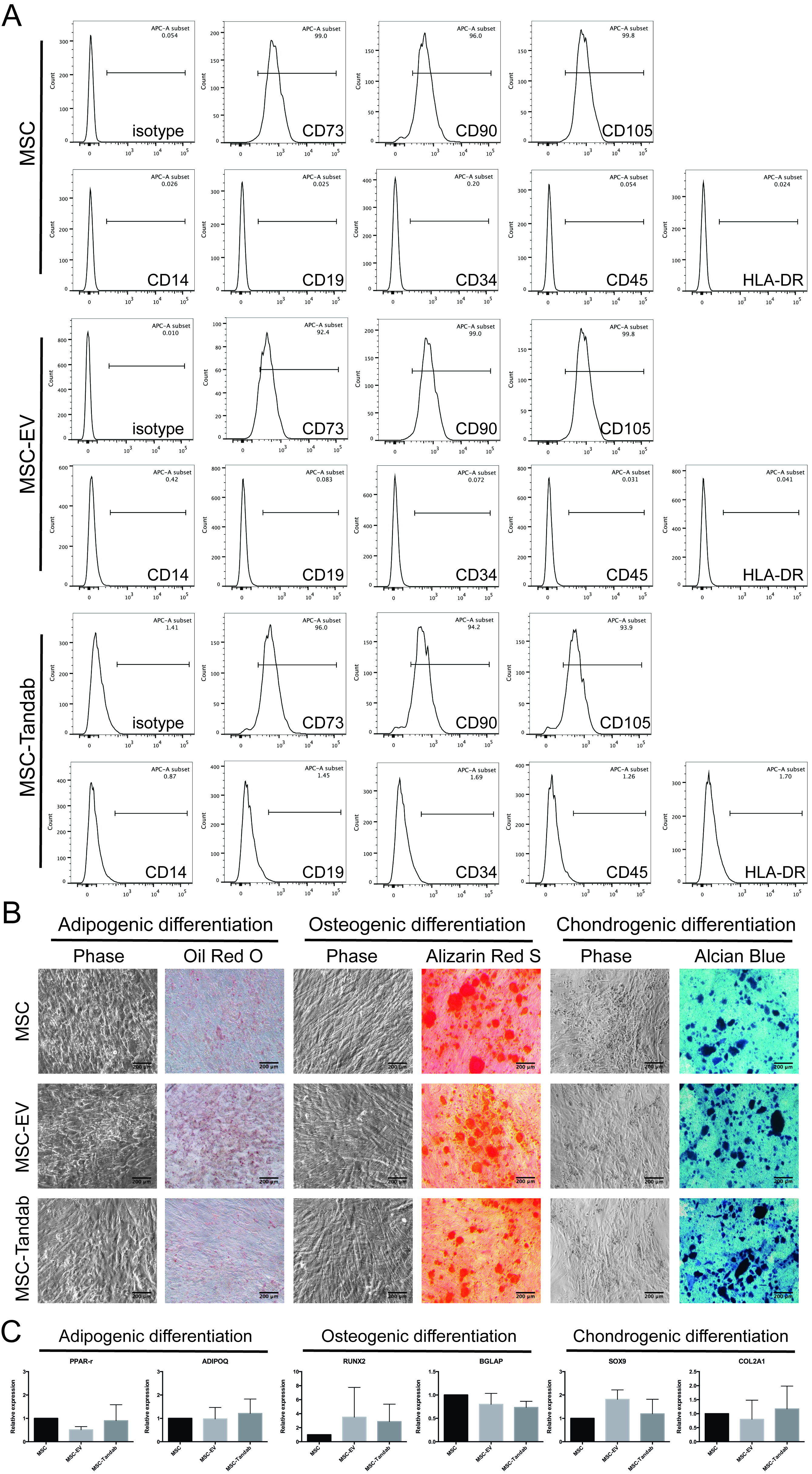

Supplement: Additional file 2: Figure S1. — Identification of MSCs and transduced MSCs. A. Phenotypic analysis of MSCs and transduced MSCs. Flow cytometric analysis showed that MSCs, MSC-EV, and MSC-Tandab expressed CD73, CD90, and CD105 but not CD14, CD19, CD34, CD45, and HLA-DR. B. Representative images showing the in vitro differentiation of MSCs and transduced MSCs into adipogenic, osteogenic, and chondrogenic lineages, respectively. Scale bar = 200 μm. C. Relative quantification of gene expression after tri-lineage differentiation in MSCs and transduced MSCs. The mRNA levels were normalized using the expression of the reference gene (GAPDH) and compared with the MSCs group. Data shown are the mean ± SD of three repeated experiments. (TIF 9594 kb) [file 13045_2017_397_MOESM2_ESM.tif]
